# Pre-hypertension and Hypertension Among University Students in Bahrain: A Study of Prevalence and Associated Risk Factors

**DOI:** 10.7759/cureus.55989

**Published:** 2024-03-11

**Authors:** Amgad E El-Agroudy, Mona Arekat, Ahmed Jaradat, Roba Hamdan, Abdullah Alnama, Ebrahim Almahmeed, Ahmad AlShammari, Reem Alanazi, Hamza Juhmani, Abrar Almarzooq

**Affiliations:** 1 Internal Medicine, Arabian Gulf University, Manama, BHR; 2 Family and Community Medicine, Arabian Gulf University, Manama, BHR

**Keywords:** students, public health hypertension, university, pre-hypertension, risk factors

## Abstract

Background/Objectives: In the last 20 years, hypertension has become more common among younger age groups. Based on a global meta-analysis, the combined prevalence of hypertension and prehypertension were 4.0% and 9.7%, respectively. This study aimed to evaluate the prevalence of prehypertension and hypertension among university students and their associated risk factors.

Methods: Four hundred and eleven students aged between 18 and 25 (196 males and 215 females) were randomly selected to participate from the College of Medicine and Medical Sciences (CMMS) and the College of Business Administration, Bahrain. The data was collected through a structured questionnaire, which gathered information about lifestyle habits. Trained students measured the participant's blood pressure and body mass index (BMI) according to standardized settings. All risk factors were studied according to the study field and their gender.

Results: The mean age of the participants was 16.4±0.9 years. Of the total participants, 61.3% (n= 252) were normotensive, 30.7% (n= 126) were pre-hypertensive, and 8% (n= 33) were hypertensive. The prevalence of hypertension and pre-hypertension was higher in male students, 13.8% (n=27) and 44.9% (n= 88), compared to female students, 2.8% (n=6) and 17.7% (n=38), respectively. The results of the univariate analysis showed an association of hypertension with the field of study, gender, age, BMI, exercise frequency, frequency of eating junk food, and family history of hypertension (p < 0.05). Multivariate logistic regression analysis found a significant association between hypertension and pre-hypertension with gender, the field of study, and BMI.

Conclusions: The findings of the study revealed that hypertension and pre-hypertension are common among university students in Bahrain. The risk factors for these conditions include studying medicine, being male, and being obese.

## Introduction

Hypertension is a condition where blood pressure remains consistently elevated. Hypertension refers to sustained high blood pressure (BP) as reported by the Eighth Joint National Committee (JNC-8), with systolic blood pressure (SBP) of 140 or more, or diastolic blood pressure (DBP) of 90 or more. Pre-hypertension refers to an SBP of 120-139 mmHg or a DBP of 80-89 mmHg. Normal BP refers to an SBP of 90-119 mmHg and a DBP of 60-79 mmHg. Hypotension refers to an SBP below 90 mmHg and a DBP below 60 mmHg. It is a growing public health concern, affecting over one billion people worldwide. It is the leading modifiable risk factor for premature death worldwide. Between 1990 and 2019, the latest global trend saw the absolute number of people with hypertension double, with two-thirds of cases found in low- and middle-income nations. This is partly due to a higher prevalence of risk factors in those populations over the past few decades [1,2].

In the last 20 years, hypertension has become more common among younger age groups. Based on a global meta-analysis, the combined prevalence of hypertension and pre-hypertension were 4.0% and 9.7%, respectively. In cases of primary hypertension, adolescents are affected up to 85-90% of the time [2,3]. Studies conducted outside the Gulf region reported variable rates of hypertension among medical students, ranging from 6% to 39% [4-6]. One study found that 8.1% of university students had high-normal arterial pressure, while the rate of hypertension was 5.6%. Other studies found rates of hypertension of 7.4% in Ethiopia [7], 19.3% in Uganda, 35.1% in Tunisia, and 33% in Ecuador [8]. In a study from Saudi Arabia [9], 7.5% of students were hypertensive, while in Jeddah, other studies reported a prevalence of 9.3% among medical students [10,11]. Finally, one study found a hypertension rate of 7% in Kuwaiti students [12].

With the rise of urbanization, socio-economic and lifestyle changes from traditional to modern ways have resulted in physical inactivity, smoking, excessive use of technology, eating excessive junk food, and high fat and calorie consumption. This has led to an increase in cases of obesity, pre-hypertension, and hypertension among the youth [13].

This study aimed to determine the prevalence of pre-hypertension and hypertension and the associated risk factors among university students.

## Materials and methods

Design and sample

A cross-sectional study was conducted on students at the College of Medicine and Medical Sciences (CMMS) at Arabian Gulf University and the College of Business Administration at Bahrain University. The study included all students in the basic and clinical years (first to fifth year). Study sample size was calculated with a confidence level of 95%, p=0.5 and margin of error 0.05 using simple sample size formula: n=(Z2_(1-α/2.P(1-P))^2)/E^2 =(1.96)^2*0.5*0.5)/0.05^2 =384

Where Z = Critical Value (1.96) and E = Margin of error (0.05)

According to these calculations, 425 students were invited to participate in the study, of whom 411 students provided complete responses.

Inclusion and exclusion criteria

The study involved selecting students who met certain eligibility criteria, such as being between 18 and 25, and being willing to participate after signing an informed consent form. Exclusion criteria included students with known chronic diseases like diabetes mellitus, hypertension, bronchial asthma, or having hereditary disease.

Ethical considerations

We obtained approval from the Ethical Committee of the Institutional Review Board (IRB) at Arabian Gulf University (E21-PI-2-12), and all students signed a consent form according to the Helsinki Declaration.

Data collection

A survey was conducted to gather information from students to collect the following details: (1) Sociodemographic data such as age, gender, nationality, educational year, and home residence; (2) Family history of hypertension and diabetes mellitus; (3) Lifestyle factors, including (a) dietary habits such as the intake of salty and junk foods; (b) smoking status, including the number of cigarettes per day, smoking duration, and other forms of tobacco use like waterpipe smoking (smoking habits were classified as ‘‘heavy’’ (over a year or more than 10 cigarettes a day), ‘‘light’’ (less than a year or less than 10 cigarettes per day), or ‘‘none’’ (nonsmoker)); and (c) physical activity, including type and frequency of physical activity. Levels of exercise were classified as ‘‘high’’ (regularly scheduled exercise), ‘‘low’’ (only the weekly 90-minute physical education classes at university), or ‘‘none’’. The survey also collected information on sleeping duration, i.e., the average number of hours of sleep in 24 hours, with 8 hours of sleep as a reference.

Measurements

All measures were done according to standardized methods.

(1) BP was measured following the American Heart Association guidelines. Trained medical students conducted the measurements in a quiet room after the subject had rested in a seated position for five minutes without consuming cigarettes, coffee, or tea. During the measurement, the subject's right arm was placed at the same level as their heart to ensure accuracy. The BP was measured using a mercury sphygmomanometer with an appropriate-size cuff. We used the ideal cuff bladder for all students (length is ≥ 80% of the patient’s arm circumference and bladder width is ≥ 40% of the patient’s arm circumference). Two recordings for each subject were taken, with a 10-minute interval between each recording. The final reading was the average of the two recordings. According to the Joint National Committee (JNC) 8 report, BP categories are defined as follows: Hypertension refers to sustained high BP, with SBP of 140 or more, or DBP of 90 or more. Pre-hypertension refers to an SBP of 120-139 mmHg or a DBP of 80-89 mmHg. Normal BP refers to an SBP of 90-119 mmHg and a DBP of 60-79 mmHg. Hypotension refers to an SBP below 90 mmHg and a DBP below 60 mmHg.

(2) We measured the weight and height of each student and then calculated their BMI by dividing the weight in kilograms by the square of their height in meters. According to the World Health Organization criteria, the BMI was categorized where a BMI below 25 kg/m^2^ is considered normal, a BMI between 25 and 29 kg/m^2^ is considered overweight, a BMI of 30 kg/m^2^ or more is considered obese, and those with a BMI below 25 kg/m^2^ are considered underweight.

Statistical analysis

The Statistical Package for the Social Sciences (IBM SPSS Statistics for Windows, IBM Corp., Version 25.0, Armonk, NY) was used to analyze the data collected in this study. Descriptive statistics such as percentage, frequency, and mean ± standard deviation (SD) were used to analyze the data. To compare the means of quantitative data, Student's t-test was used, while categorical data were compared using the chi-square “χ2” test. The study also conducted univariate and multivariate logistic regression analyses to determine the association between gender, age, BMI, nutritional lifestyle factors like salt intake and fast-food intake, physical activity, sleep duration, smoking pattern, family history of hypertension, and diabetes mellitus. All analyses were conducted at a 95% confidence level. A p-value of less than 0.05 was deemed statistically significant.

## Results

The study had 425 participants, all of whom were from Arabian Gulf Nationalities. The response rate among students who provided complete responses was 96.5% (n= 411). The age was between 18 and 25 years, with an average age of 20.8 years (±1.83). More than half of the participants (n=215,52.3%) were females, with the majority being from Bahrain (n=138, 38.4%). Regarding academic years, (n=137, 33.3%) were in their fifth and sixth years. Table 1 shows detailed information on the demographic characteristics of the participants according to gender and their field of study. Most participants in the College of Medicine were males (53.5%), while females made up the majority in Business Administration (63.9%). Most participants in both fields of study were 20 or younger (n= 206, 50.1%).

**Table 1:** Demographic Characteristics of the Participants in the Study According to Gender and Field of Study Data represented as N and %. Differences with P <0.05 were considered statistically significant and 0.0001 highly significant.

		Male (n=196)			Female (n=215)						
		n	%	n		%	Total		P-Value		
Field of study	Medical	147	53.5	128		46.5	275		0.001		
	Non-Medical	49	36.0	87		64.0	136				
Age/Years	18-20	85	41.3	121		58.7	206		0.0001		
	21-23	89	49.7	90		50.3	179				
	> 23	22	84.6	4		15.4	26				
Level	Year 1	16	34.8	30		65.2	46		0.004		
	Year 2	28	41.2	40		58.8	68				
	Year 3	41	44.6	51		55.4	92				
	Year 4	36	52.9	32		47.1	68				
	Year 5	22	39.3	34		60.7	56				
	Year 6	53	65.4	28		34.6	81				
Nationality	Bahraini	57	41.3	81		58.7	138		0.0001		
	Saudi	99	62.7	59		37.3	158				
	Kuwaiti	30	32.3	63		67.7	93				
	Others	9	33.3	12		66.7	21				

		Medical (n=275)		Non-medical (n=136)		Total	P-Value
		n	%	n	%		
Gender	Male	147	75.0	49	25.0	196	0.001
	Female	128	59.5	87	40.5	215	
Age/Years	18-20	123	59.7	83	40.3	206	0.006
	21-23	131	73.2	48	26.8	179	
	> 23	21	80.8	5	19.2	26	
Level	Year 1	26	56.5	20	43.5	46	0.032
	Year 2	41	60.3	27	39.7	68	
	Year 3	56	60.9	36	39.1	92	
	Year 4	48	70.6	20	29.4	68	
	Year 5	46	82.1	10	17.9	56	
	Year 6	58	71.6	23	28.4	81	
Nationality	Bahraini	96	69.6	42	30.4	138	0.487
	Saudi	103	65.2	55	34.8	158	
	Kuwaiti	58	62.4	35	37.6	93	
	Others	18	85.7	3	14.3	21	

According to the JNC-8 definition of hypertension and pre-hypertension, most (n = 252, 61.3%) had BP within the normal range (median systolic BP 113.6 ± 8.3 mmHg and diastolic BP 72.1 ± 9.7 mmHg). In comparison, (n = 126, 30.7%) were pre-hypertensive (median systolic BP 129.5 ± 7.5 mmHg and diastolic BP 81.3 ± 9.3 mmHg), and 8% (n = 33) had BP in the hypertensive range (median systolic pressure 143.0 ± 12.4 mmHg and diastolic BP 89.5 ± 10.5 mmHg).

Table 2 presents the outcomes of the univariate analysis on hypertension, pre-hypertension, and various risk factors. The results indicate that medical students have a significantly higher percentage of pre-hypertension (n= 95, 34.5%) and hypertension (n= 26, 9.5%) compared to non-medical students (n= 31, 22.8% and n= 7, 5.1%), respectively. Moreover, male students (n=88,44.9%) and older students (n=12, 46.2%) with pre-hypertension had statistically significant differences compared to students with normal BP. This trend was also observed among male hypertensive students (n=27, 13.8%) and older students (n=5, 19.2%). Additionally, Saudi students have a higher percentage of hypertension compared to other nationalities, with a statistically significant difference (P=0.0001).

**Table 2:** Univariate Analysis of Risk Factors Associated With Pre-Hypertension and Hypertension in All Studied Groups Data represented as N and %. Differences with P <0.05 were considered statistically significant and 0.0001 highly significant. BMI: Body mass index

				Normal										Prehypertensive					Hypertensive					
			n								%		n		%	P-value				n	%	P-value		
Field of study		Medical				154					56.0		95		34.5	0.006		26			9.5	0.048		
		Non-medical								98		72.1	31		22.8			7			5.1			
Gender		Male	81								41.3		88		44.9	0.0001		27			13.8	0.0001		
		Female	171								79.5		38		17.7			6			2.8			
Age/Years		18-20	137								66.5		53		25.7	0.013		16			7.8	0.015		
		21-23	106								59.2		61		34.1			12			6.7			
		> 23	9								34.6		12		46.2			5			19.2			
Nationality		Bahraini			90						65.2		37		26.8	0.277		11			8.0	0.0001		
		Saudi			87						55.1		56		35.4			15			9.5			
		Kuwaiti			59						63.4		28		30.1			6			6.5			
		Others			15						71.4		5		23.8			1			4.8			
Inhabitance		Big city	135								60.0		71		31.6	0.813		19			8.4	0.832		
		Small City					108				63.2		51		29.8			12			7.0			
		Rural	8								66.7		3		25.0			1			8.3			
Family History of Hypertension		Yes	113								44.8		71		56.3	0.035		20			62.6	0.049		
		No	139								67.5		55		26.7			12			5.8			
Family History of Diabetes Mellitus		Yes	111								44.0		73		57.9	0.011		17			53.1	0.331		
		No	141								67.5		53		42.1			15			7.2			
Cigarettes Number Per Day		< = 10	11								40.7		12		44.4	0.336		4			14.8	0.659		
		> 10	7								53.8		4		30.8			2			15.4			
Cigarettes Duration		<=36	17								54.8		10		32.3	0.204		4			12.9	0.176		
		> 36	1								11.1		6		66.7			2			22.2			
Waterpipe Smoking		Yes	27								50.9		20		37.7	0.152		6			11.3	0.207		
		No	225								62.8		106		29.6			27			7.5			
Waterpipe Smoking Per Week		<=2	21								56.8		12		32.4	0.572		4			10.8	0.100		
		> 2	10								50.0		8		40.0			2			10.0			
Exercise		Yes	138								59.5		71		30.6	0.770		23			9.9	0.104		
		No	114								63.7		55		30.7			10			5.6			
Exercise Frequency Per Week		<=3	114								63.3		50		27.8	0.267		16			8.9	0.0001		
		>3	37								56.9		23		35.4			5			7.7			
Salt Intake		NO	34								70.8		12		25.0	0.668		2			4.2	0.153		
		Low	56								62.2		31		34.4			3			3.3			
		Moderate				147					59.5		76		30.8			24			9.7			
		High	15								62.5		6		25.0			3			12.5			
Sleep Duration (Hours)		< 8	150								61.0		77		31.3	0.766		19			7.7	0.830		
		>= 8	102								61.8		49		29.7			14			8.5			
Dietary Habits (Junk Food)		Yes	155								58.7		86		32.6	0.198		23			8.7	0.361		
		No	97								66.0		40		27.2			10			6.8			
Junk Food Frequency Per Week		0	77								68.1		30		26.5	0.079		6			5.3	0.0001		
		1	40								63.5		19		30.2			4			6.3			
		2	53								60.9		28		32.2			6			6.9			
		3	31								46.3		29		43.3			7			10.4			
		> 3	51								63.0		20		24.7			10			12.3			
BMI Groups		Underweight								18		85.7	3		14.3	0.0001		0			0.0	0.0001		
		Normal						176			71.8		60		24.5			9			3.7			
		Overweight						46			46.9		41		41.8			11			11.2			
		Obese (>30)							11		23.9		22		47.8			13			28.3			

Our analysis of the data shows that there was no significant difference in the proportion of students with pre-hypertension and hypertension between those who smoked cigarettes or waterpipe, regularly exercised, or consumed more salt. More than 50% of the students exercised regularly, but this percentage was lower among pre-hypertensive and hypertensive participants (n= 71, 30.6%, and n= 23, 9.9%, respectively) than normotensive ones (P=0.104). Our study included 411 students (n= 264, 64.2%) who consumed junk food and (n= 246, 59.9%) who slept less than eight hours daily. Sleep deprivation was not a statistically significant factor for pre-hypertension or hypertension. The percentage of students who did not consume junk food was 68.1% (n= 77), 26.5% (n= 30), and 5.3% (n= 6) for normotensive, pre-hypertensive, and hypertensive students, respectively. The difference between normotensive and pre-hypertensive students was not statistically significant (P=0.079), while the difference between normotensive and hypertensive students was highly statistically significant (P<0.0001). Obesity was found in 47.8% (n= 22) of pre-hypertensive students and 28.3% (n= 13) of hypertensive students. BMI was a highly statistically significant risk factor for pre-hypertension (P=0.0001) and hypertension (P=0.0001) among students.

About 49.6% of students (n=204) reported a family history of hypertension. About 44.8% (n= 113) were normotensive, while 56.3% (n= 71) were pre-hypertensive or 62.6% (n= 20) hypertensive. According to the univariate analysis, there was an association between a family history of hypertension and a higher prevalence of pre-hypertension (P=0.035) and hypertension (p=0.049). Pre-hypertensive students had a higher percentage of positive family history of diabetes mellitus (n= 73, 57.9%) compared to students with normal BP (n= 111, 44.0%) (P= 0.011), but no significant difference was observed among students with hypertension (P= 0.331).

Tables 3-4 contain a multivariate logistic regression model that analyzes the risk factors for hypertension and pre-hypertension. According to the logistic regression analysis, gender and BMI are the significant risk factors for both hypertension and pre-hypertension. The odds of having hypertension rise five times for medical students (odd = 5.668, P value = 0.019), four times for male students (odd = 4.039, P value = 0.032), and around one time (odd = 0.772, P value = 0.001) for obese students. Similarly, the odds of having pre-hypertension increase two times for medical students (odd = 2.357, P value = 0.038), six times for male students (odd = 6.432, P value <0.001), and roughly one time (odd = 0.766, P value <0.001) for obese students.

**Table 3:** Logistic Regression Analysis (Pre-hypertension Versus Normal) Variable(s) entered in step 1: Field of study, Age, Grades, Gender, SH Smoking, Shisha, Exercise, Salt intake, Sleep duration, Family History of Hypertension, weekly exercise frequency, Junk food frequency per week, BMI (Body Mass Index).

Risk Factors	S.E.	Wald	df	P value	Odds Ratio	95% C.I. for Odds Ratio	
						Lower	Upper
Field of study	0.413	4.300	1	0.038	2.357	1.048	5.298
Age	0.442	2.685	1	0.101	0.484	0.204	1.153
Grades	0.508	2.124	1	0.145	2.096	0.775	5.673
Gender	0.420	19.683	1	0.001	6.432	2.826	14.638
Waterpipe smoking	0.726	0.080	1	0.777	0.814	0.196	3.376
Exercise	0.631	0.079	1	0.778	1.195	0.347	4.118
Exercise frequency per week	0.098	0.032	1	0.858	1.018	0.840	1.232
Salt intake	0.272	0.648	1	0.421	0.803	0.471	1.370
Junk food frequency per week	0.090	0.731	1	0.392	1.080	0.905	1.288
Sleep duration	0.135	0.758	1	0.384	1.125	0.863	1.465
Family history of hypertension	0.364	1.370	1	0.242	1.531	0.750	3.125
BMI	0.052	26.009	1	0.001	0.766	0.691	0.848
Constant	8.298	3.911	1	0.048	13412763.822		

**Table 4:** Logistic Regression Analysis (Hypertension Versus Normal) Data presented as odds ratio and 95% confidence interval with p-value. Differences with P <0.05 were considered statistically significant and 0.0001 highly significant.

						95% C.I. for Odds Ratio	
Risk Factors	S.E.	Wald	df	P value	Odds ratio	Lower	Upper
Field of study	0.737	5.541	1	0.019	5.668	1.337	24.028
Age	0.646	1.171	1	0.279	0.497	0.140	1.763
Gender	0.650	4.611	1	0.032	4.039	1.130	14.442
Waterpipe smoking	0.831	0.001	1	0.972	0.971	0.191	4.949
Exercise	0.455	2.541	1	0.111	2.065	0.847	5.036
Exercise frequency per week	0.147	0.025	1	0.873	0.977	0.732	1.303
Salt intake	0.485	1.553	1	0.213	0.547	0.212	1.413
Junk food frequency per week	0.154	1.223	1	0.269	1.185	0.877	1.603
Sleep duration	0.211	0.443	1	0.506	0.869	0.574	1.315
Family history of hypertension	0.600	0.001	1	0.975	0.981	0.303	3.181
BMI	0.081	10.091	1	0.001	0.772	0.658	0.906
Constant	8375.155	0.000	1	1	0.077		

## Discussion

In the past two decades, the prevalence of hypertension has increased progressively among younger people [1,3]. This study was conducted to determine the prevalence of pre-hypertension and hypertension and the risk factors among university college students in Bahrain. The study found that 30.7% of the participants had pre-hypertension; this is similar to the prevalence of 27.1% in a university in Palestine [14] and 23.3% in Jordan [15]. However, the prevalence of pre-hypertension among undergraduate students was higher in some other countries, like 56% at one university in Saudi Arabia [16], 39% at a Sudanese university [17], and 39.5% in Kuwait [12], and a lower prevalence among other Iraqi students (17.2%) [18]. The prevalence of hypertension among the participants was 8%, which is similar to the study conducted on Moroccan students which revealed the prevalence of hypertension was 9.6% [19], and the study done by Baig et al. [9] who reported 9.3% among medical students in Jeddah and 11.8% in college students from Kuwait [12], but lower than 16.7% and 26.5% from two studies done in Egypt [20,21]. In the Middle East region, the prevalence of hypertension varies widely from population to population depending on biological, demographic, social, and environmental factors present in each of them. Differences in prevalence rates in these studies also could be due to differences in the age groups constituting the study populations investigated, different cut-off points in determining the level of hypertension, socio-cultural differences, sampling methods, study settings, and timeframes of the studies.

The study found that gender played a significant role in the incidence of hypertension, with a higher proportion of male students (13.8%) being hypertensive compared to female students (2.8%); the odds of having hypertension rise four times for male students. The same trend was seen among students with pre-hypertension, with 44.9% of male students and 17.7% of female students being affected. Other studies [8,12,19-21] have also observed this trend. This gender difference could be the variations in hormonal activity between males and females.

Our research found that medical students had a much higher incidence of hypertension and pre-hypertension, at rates of 78.8% and 75.4%, respectively, compared to non-medical students, whose rates were 21.2% and 24.6%; the odds of having hypertension rise five times for medical students (odd = 5.668, P value = 0.019). This is consistent with the findings of numerous studies on hypertension among medical students [6,22]. Medical students have a 2.4 times higher risk of stage 2 hypertension than young adults aged 18-39, according to Mok et al. [22]. The stress of intense medical training, in addition to anxiety, lack of exercise, insufficient sleep, and/or poor diet, may be contributing factors. One study found that hypertension and depression were particularly common among medical students [23].

Overweight and obesity, along with other lifestyle habits such as lack of exercise, smoking, excessive salt, and junk food consumption, have been identified as major contributors to pre-hypertension and hypertension development [8,12,19,20-22]. Our study found a strong association between higher BMI and hypertension prevalence among university students. The data indicated that students with a BMI of 30 or higher had the highest percentage of hypertension (39.4%), followed by pre-hypertensive students (17.5%) and normotensive students (4.4%) (P <0.001). These results are in line with previous research by ElWabel et al. [8] and Mehdad et al. [19]. While the exact process through which higher body weight causes an increase in BP is not completely understood, previous studies indicate that a combination of insulin resistance, elevated leptin levels, and heightened sympathetic activity are the main culprits in obese individuals.

The present study reveals that the risk of developing pre-hypertension is not significantly associated with many reported risk factors like exercise practice, high salt intake, and smoking. This finding of a nonsignificant association of smoking was not in agreement with the results of previous studies by Ibrahim et al. [24] and Sundar et al. [25], who also reported that an increased risk of hypertension was directly associated with an increase in daily cigarette smoking. Our study found a non-significant association by regression analysis between the frequency of physical exercise and hypertension which contradicts the results in the study conducted by Moussa et al. [21]. Our results were not in line with Sundar et al. [25] who found that physical activity has no significant relationship with hypertension in Indian students. Regarding sleep patterns, some research suggests that the increasing prevalence of hypertension could be related to decreased sleep time. Several studies have shown that BP and sympathetic nervous system activity significantly increase when sleep is restricted to less than six hours, both for normotensive and hypertensive individuals [26]. However, in this study, sleeping hours were not statistically associated with BP levels. While adding extra salt to one's diet was identified as a significant risk factor in a study conducted in New Delhi [26], it was not seen as significant in our study, which is consistent with a study conducted by Parsekar et al. [27].

The data presented in this study indicates that college students who have a family history of hypertension are at higher risk of developing pre-hypertension (56.8%) and hypertension (62.5%) by univariate analysis, but not evident by regression analysis. These findings are in line with many studies that have shown a strong association between family history and hypertension [18,21,28]. Moussa et al. [21] demonstrated that having a family history of hypertension doubles the risk of developing hypertension. Therefore, it is crucial to screen family members, particularly those with a family history of hypertension, to identify those at risk.

Limitations

There are some limitations of this study when interpreting the findings. First, BP was only measured on a single visit, and only two were taken. However, most epidemiological studies of hypertension in adolescents also relied on single-visit readings. Secondly, the study relied on a written questionnaire for information on lifestyle such as sleeping, junk food and salt consumption, smoking, and exercise, which are subject to recall bias.

## Conclusions

The study found that a significant number of undergraduate students have pre-hypertension and hypertension. The risk factors for these conditions include studying medicine, male gender, and obesity. These findings highlight the need for lifestyle modification programs to be incorporated into the university's curriculum to prevent the progression of hypertension. Additionally, there should be plans to treat hypertensive students. A comprehensive approach is necessary to prevent the disease by detecting it early among university students through regular medical check-ups that measure BP.
